# Multimodal Treatment for Female Primary Urethral Carcinoma: A Three‐Case Series Utilizing Robot‐Assisted Radical Cystectomy and Extended Resection

**DOI:** 10.1002/iju5.70229

**Published:** 2026-07-26

**Authors:** Yuya Oishi, Hiromitsu Watanabe, Shinya Watanabe, Kyohei Watanabe, Yuto Matsushita, Keita Tamura, Daisuke Motoyama, Teruo Inamoto

**Affiliations:** ^1^ Department of Urology Hamamatsu University School of Medicine Hamamatsu Japan; ^2^ Department of Urology JA Shizuoka Kohseiren Enshu Hospital Hamamatsu Japan; ^3^ Department of Urology Chutoen General Medical Center Kakegawa Japan; ^4^ Department of Developed Studies for Advanced Robotic Surgery Hamamatsu University School of Medicine Hamamatsu Japan

**Keywords:** extended resection, multimodal treatment, primary urethral carcinoma, robot‐assisted radical cystectomy, urethrectomy

## Abstract

**Introduction:**

Primary urethral carcinoma (PUC) is a rare malignancy, particularly in females, with no established standard treatment. We herein examined the clinical outcomes of three female patients who underwent multimodal therapy with robot‐assisted radical cystectomy (RARC) and extended surgical resection.

**Case Presentation:**

Three female patients (45, 65, and 71 years) were diagnosed with PUC (adenocarcinoma, cT3N0M0; clear cell carcinoma, cT3N2M0; and squamous cell carcinoma, cT4N0M0). Two patients received cisplatin‐based neoadjuvant chemotherapy. All patients underwent RARC with en bloc urethrectomy and extended resection of the anterior vaginal wall or pelvic fascia. One achieved a pathological complete response, one developed local recurrence due to a positive surgical margin, and two remained recurrence‐free.

**Conclusion:**

Individualized multimodal strategies combining perioperative chemotherapy and extended surgical resection are crucial for managing locally advanced PUC in females.

## Keynote Message

Primary urethral carcinoma in females is rare and often advanced at diagnosis. This case series demonstrates that a multimodal approach, integrating cisplatin‐based neoadjuvant chemotherapy with RARC and extended resection, may achieve favorable local control. Surgeons should consider aggressive en bloc resection of adjacent organs, such as the anterior vaginal wall and endopelvic fascia, to ensure a negative surgical margin.

**Abbreviations**
AC: adenocarcinoma
CBDCA: carboplatin
CCC: clear cell carcinoma
CDDP: cisplatin
CR: complete response
GEM: gemcitabine
LOS: length of stay
MIS: minimally invasive surgery
MRI: magnetic resonance imaging
NAC: neoadjuvant chemotherapy
OS: overall survival
PR: partial response
PTX: paclitaxel
PUC: primary urethral carcinoma
RARC: robot‐assisted radical cystectomy
SCC: squamous cell carcinoma
SD: stable disease
UC: urothelial carcinoma

## 1 Introduction

PUC accounts for less than 1% of all urological malignancies [1]. The age‐standardized incidence per 1 000 000 is higher in males than females (2.70 vs. 0.55) and has remained stable, with a male‐to‐female ratio of approximately 4.9:1 [2]. Histological distributions also vary by sex; while urothelial carcinoma (UC) is common in males, females show a higher prevalence of AC (38%–46.7%) and SCC (25.4%–28%) [3, 4, 5]. Due to its rarity, no prospective clinical trials or standardized treatments have been established. We herein report three cases of female PUC treated with RARC and extended surgical resection as part of a multimodal approach.

## 2 Case Presentations

### 2.1 Case 1

**FIGURE 1:**
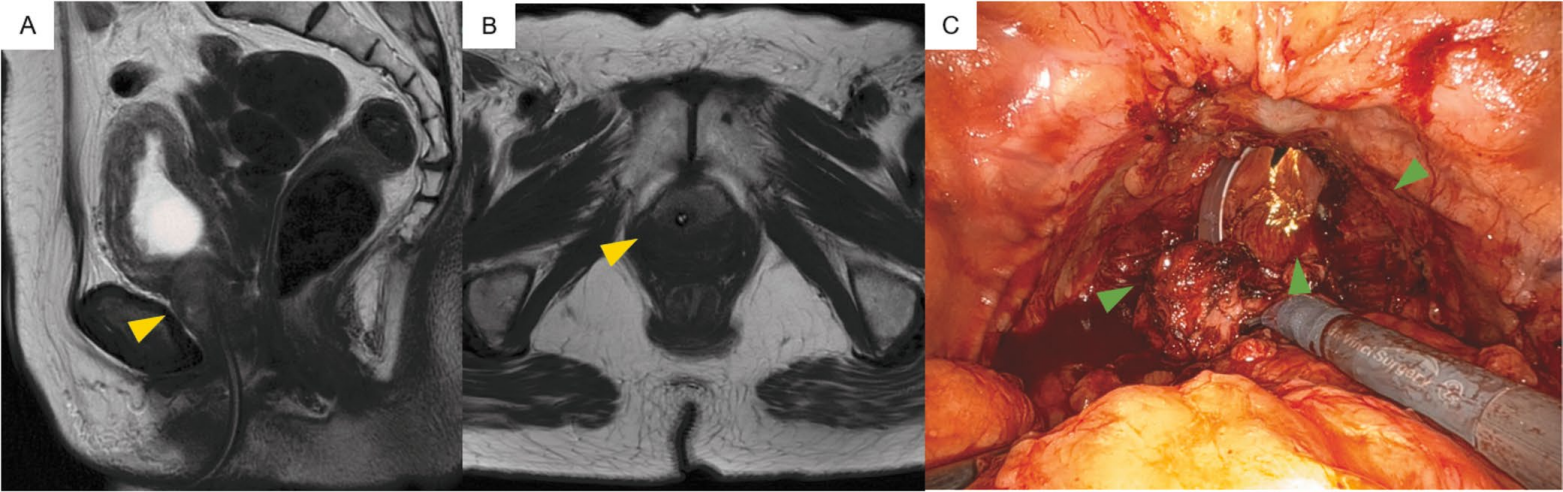
Sagittal (A) and axial (B) T2‐weighted magnetic resonance images of Case 1 before chemotherapy. The yellow arrows indicate a 23‐mm mass in the urethral diverticulum. Tumor invasion into the anterior vaginal wall was suggested. (C) The green arrows indicate that the anterior vaginal wall, external urethral meatus, and levator ani muscle within the endopelvic fascia were resected en bloc.

A 71‐year‐old female presented with difficulty urinating. T2‐weighted MRI showed a mass arising within the urethral diverticulum with invasion into the anterior vaginal wall (Figure 1). Biopsy revealed AC (cT3N0M0). NAC consisting of GEM + CBDCA was administered for three cycles. Because no standard neoadjuvant chemotherapy regimen has been established for primary urethral adenocarcinoma, and more intensive chemotherapy for adenocarcinoma was considered difficult because of the patient's age, renal function, and preoperative setting, GEM + CBDCA was selected based on treatment strategies used for advanced urothelial carcinoma and treatment tolerability. The best response was SD. She subsequently underwent RARC with pelvic lymph node dissection including the external iliac, internal iliac, obturator, and presacral lymph nodes, combined with en bloc urethrectomy and extended resection of the anterior vaginal wall and endopelvic fascia (Figure 1). A pathological examination revealed ypT3N0 disease with a positive surgical margin at the anterior vaginal wall. No adjuvant therapy was administered. Local recurrence at the vaginal stump was detected at 18 months after surgery and was treated with radiation therapy. She remains alive 44 months after surgery.

### 2.2 Case 2

**FIGURE 2:**
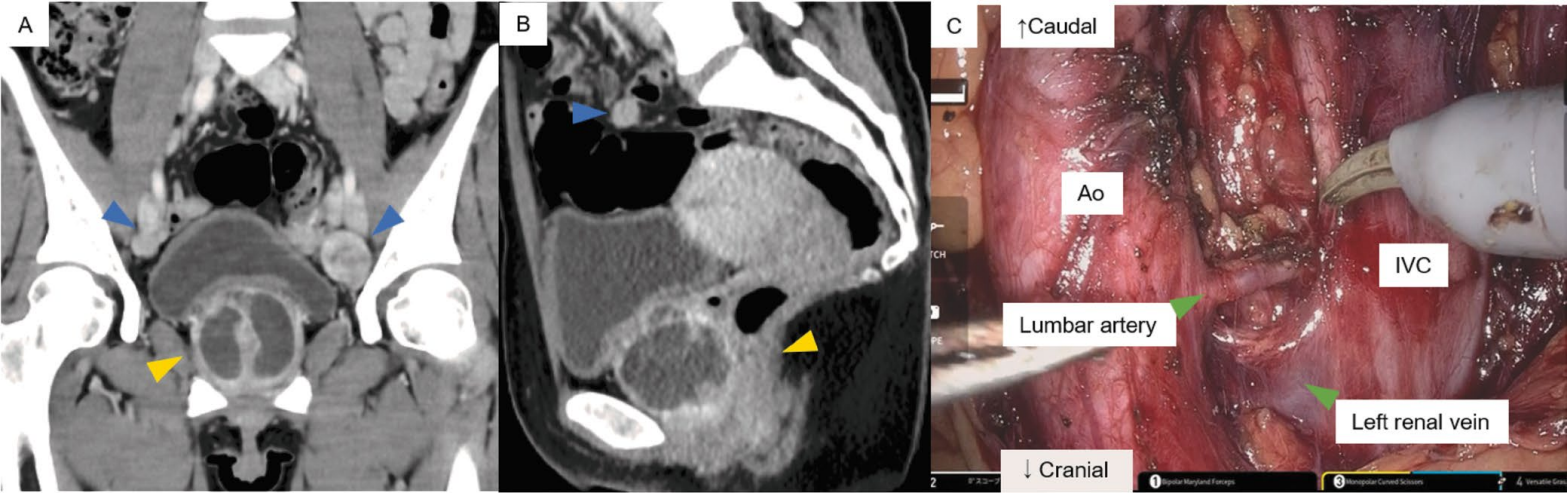
Sagittal (A) and coronal (B) contrast‐enhanced computed tomography images of Case 2 preoperatively. The yellow arrows indicate a 45 mm × 42 mm × 48 mm cystic tumor in the urethra, and the blue arrows indicate bilateral obturator and common‐iliac lymph node enlargement. (C) A super‐extended lymph node dissection, including para‐aortic lymph node, was performed. The green arrows indicate the lumbar artery and the left renal vein, respectively. Ao, aorta; IVC, inferior vena cava.

A 45‐year‐old female was incidentally found to have a urethral mass on imaging. Contrast‐enhanced computed tomography showed a cystic urethral mass and bilateral obturator and common‐iliac lymph node enlargement (Figure 2). She was diagnosed with CCC (cT3N2M0). Due to the lack of effective regimens, she underwent RARC, urethrectomy combined with anterior vaginal wall and endopelvic fascia resection, and super‐extended lymph node dissection (pelvic and para‐aortic) without NAC (Figure 2). A pathological examination revealed pT3N2 disease with a negative surgical margin. No adjuvant therapy was administered. She remains free of recurrence 24 months after surgery.

### 2.3 Case 3

**FIGURE 3:**
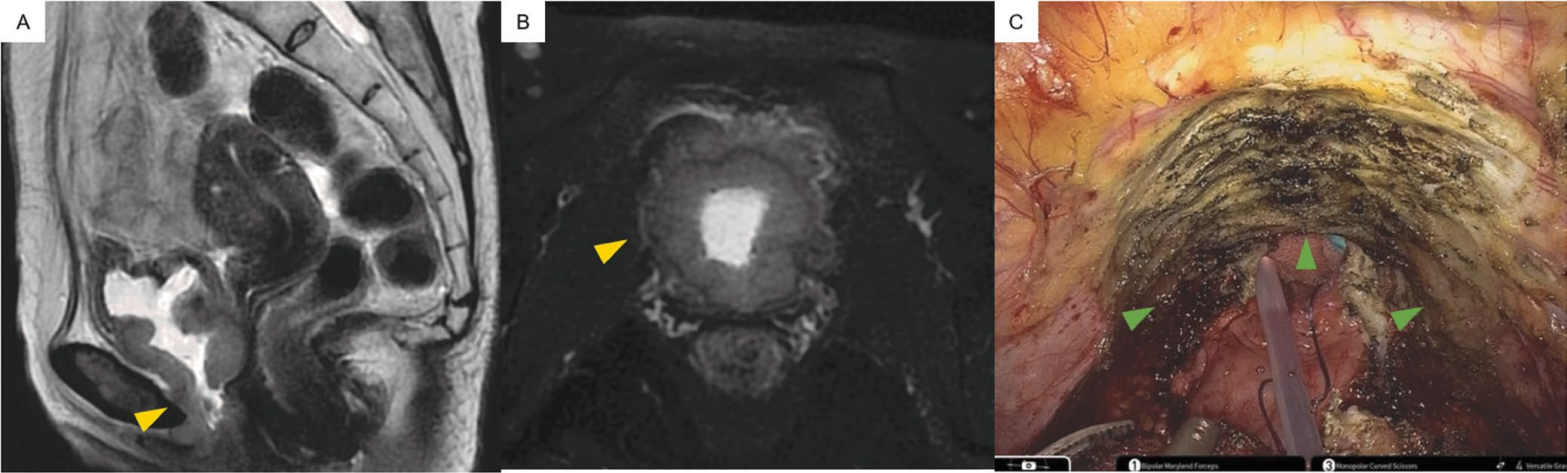
Sagittal (A) and axial (B) T2‐weighted magnetic resonance images of Case 3 before chemotherapy. The yellow arrows indicate a mass extending from the bladder neck to the external urethral meatus, with irregular wall thickening and urethral dilatation. The tumor was in close proximity to the levator ani muscle, pubic bone, and vaginal wall, suggesting invasion. (C) The green arrows indicate that the pubic periosteum and the levator ani muscle were resected en bloc.

A 65‐year‐old female presented with hematuria and urinary incontinence. T2‐weighted MRI showed a mass with irregular wall thickening and urethral dilatation, suggesting invasion into the levator ani muscle, pubic bone, and vaginal wall (Figure 3). Biopsy revealed SCC (cT4N0M0). NAC consisting of PTX + CDDP was administered for three cycles, and the best response was PR. She subsequently underwent RARC with pelvic lymph node dissection including the external and internal iliac lymph nodes and extended resection of the anterior vaginal wall, levator ani muscle, and pubic periosteum (Figure 3). A pathological examination revealed pathological CR (ypT0N0) with a negative surgical margin. No adjuvant therapy was administered. She remains free of recurrence 14 months after surgery.

The clinical characteristics of the three cases are summarized in Table 1.

**TABLE 1:** Clinical characteristics.

	Case 1	Case 2	Case 3
Age (years)	71	45	65
Histology	AC	CCC	SCC
Clinical stage	cT3N0M0	cT3N2M0	cT4N0M0
Neoadjuvant chemotherapy (cycles)	GEM + CBDCA (3)	None	PTX + CDDP (3)
Best overall response	SD	None	PR
Pathological stage	ypT3N0	pT3N2	ypT0N0
Surgical margin	Positive	Negative	Negative
Follow‐up (months)	44	24	14
Oncological outcome	Local recurrence	Recurrence‐free	Recurrence‐free

Abbreviations: AC, adenocarcinoma; CBDCA, carboplatin; CCC, clear cell carcinoma; CDDP, cisplatin; GEM, gemcitabine; PR, partial response; PTX, paclitaxel; SCC, squamous cell carcinoma; SD, stable disease.

## 3 Discussion

PUC is a rare malignancy that is characterized by diverse histological types [1]. A higher proportion of female patients present with locally advanced disease or lymph node metastasis at diagnosis, which leads to a poorer prognosis [6]. The 5‐year OS rates of female PUC patients who underwent surgery or received radiation therapy were 67%, 53%, and 17% for stages 0–II, III, and IV, respectively, while 5‐year survival rates for SCC, UC, and AC were 64%, 61%, and 31%, respectively [7]. To manage locally advanced cases, a multimodal treatment approach is currently considered standard.

A SEER database study of 1544 PUC patients showed significantly higher 5‐year OS rates with local therapy (39.8%) or radical surgery (44.7%) compared to no surgery (21.5%, *p* < 0.001). Furthermore, a multivariate analysis identified primary site surgery as an independent predictor of prolonged OS (local therapy, *p* = 0.037; radical surgery, *p* < 0.001) and reduced cancer‐specific mortality (*p* = 0.003) [8].

Guidelines from the European Association of Urology recommend radical excision for proximal urethral tumors or advanced disease in females. This includes en bloc removal of the bladder and urethra with the anterior vaginal wall, bilateral bulbocavernosus muscles, and soft tissue adjacent to the pubic symphysis, in addition to pelvic lymph node dissection and, if palpable, inguinal lymph node dissection [9]. In our series, preoperative imaging demonstrated suspected invasion into the bladder neck, making it clinically difficult to distinguish these tumors from primary bladder cancer with distal urethral extension. Furthermore, because of the histological heterogeneity of these tumors, a definitive pathological distinction between pure primary urethral carcinoma and primary bladder urothelial carcinoma with variant histology may be challenging in locally advanced cases. Therefore, we performed RARC with extended resection according to the surgical strategy used for muscle‐invasive bladder cancer, which is a standard indication for robotic surgery in Japan. Robot‐assisted surgery may facilitate en bloc resection of tumors located deep within the pelvis. Moreover, RARC may be more favorable than open surgery in terms of minimizing blood loss and blood transfusion, promoting a shorter LOS, and reducing the high risk of thromboembolic events [10]. MIS may reduce perioperative complications associated with extended resection. To our knowledge, there have been no previous reports describing robotic radical surgery for female PUC. We consider robot‐assisted surgery to be a feasible treatment option for female patients with PUC.

Recent studies suggest that the combination of CDDP‐based NAC followed by surgery prolongs both OS and recurrence‐free survival. In a study of 124 patients (including 38 females), the 3‐year OS rate was 100% in patients achieving an objective response (CR or PR) to neoadjuvant therapy, compared with 58.3% in those with SD or progressive disease [11]. Thus, a favorable response to NAC contributes to improved survival outcomes.

It is difficult to select an optimal regimen for urethral cancer due to histological diversity. In this series, case 3 achieved pathological CR following neoadjuvant PTX + CDDP, whereas case 1 developed local recurrence with a positive surgical margin after GEM + CBDCA. These results indicate the potential of appropriately selected NAC to facilitate tumor downstaging and enhance local control during radical resection, while highlighting the limited response to chemotherapy, which suggests the need for further perioperative strategies, including the integration of radiotherapy.

## 4 Conclusion

Multimodality treatment, including NAC, extensive surgical resection, and radiotherapy, is essential to improve outcomes in female patients with locally advanced PUC. In addition, robotic surgery is a treatment option to consider when planning surgical strategy for PUC.

## Ethics Statement

This study was approved by the Institutional Review Board of Hamamatsu University School of Medicine (approval No. 21‐090). In addition, the surgical procedure was reviewed and approved by the High‐Difficulty New Medical Technology Review Committee of Hamamatsu University School of Medicine (approval No. H281110).

## Consent

Informed consent was obtained from all subjects.

## Conflicts of Interest

The authors declare no conflicts of interest.

## Data Availability

The data that support the findings of this study are available on request from the corresponding author. The data are not publicly available due to privacy or ethical restrictions.

## References

[1] G. Gatta , J. M. van der Zwan , P. G. Casali , et al., “Rare Cancers Are Not So Rare: The Rare Cancer Burden in Europe,” European Journal of Cancer 47, no. 17 (2011): 2493–2511, 10.1016/j.ejca.2011.08.008. 22033323

[2] M. Wenzel , L. Nocera , C. Collà Ruvolo , et al., “Incidence Rates and Contemporary Trends in Primary Urethral Cancer,” Cancer Causes & Control 32, no. 6 (2021): 627–634, 10.1007/s10552-021-01416-2. 33751293 PMC8089076

[3] F. Rabbani , “Prognostic Factors in Male Urethral Cancer,” Cancer 117, no. 11 (2011): 2426–2434, 10.1002/cncr.25787. 24048790

[4] I. Aleksic , S. Rais‐Bahrami , M. Daugherty , P. K. Agarwal , S. Vourganti , and G. Bratslavsky , “Primary Urethral Carcinoma: A Surveillance, Epidemiology, and End Results Data Analysis Identifying Predictors of Cancer‐Specific Survival,” Urology Annals 10, no. 2 (2018): 170–174, 10.4103/UA.UA_136_17. 29719329 PMC5907326

[5] W. Sui , A. RoyChoudhury , S. Wenske , G. J. Decastro , J. M. McKiernan , and C. B. Anderson , “Outcomes and Prognostic Factors of Primary Urethral Cancer,” Urology 100 (2017): 180–186, 10.1016/j.urology.2016.09.042. 27720774

[6] M. Wenzel , L. Nocera , C. Collà Ruvolo , et al., “Sex‐Related Differences Include Stage, Histology, and Survival in Urethral Cancer Patients,” Clinical Genitourinary Cancer 19, no. 2 (2021): 135–143, 10.1016/j.clgc.2020.12.001. 33526327

[7] J. W. Derksen , O. Visser , G. B. de la Rivière , E. J. Meuleman , E. A. Heldeweg , and B. W. Lagerveld , “Primary Urethral Carcinoma in Females: An Epidemiologic Study on Demographical Factors, Histological Types, Tumour Stage and Survival,” World Journal of Urology 31, no. 1 (2013): 147–153, 10.1007/s00345-012-0882-5. 22614443

[8] J. Wu , Y. C. Wang , W. J. Luo , D. Bo , D. W. Ye , and Y. P. Zhu , “Primary Tumor Surgery Improves Survival in Non‐Metastatic Primary Urethral Carcinoma Patients: A Large Population‐Based Investigation,” BMC Cancer 21, no. 1 (2021): 857, 10.1186/s12885-021-08603-z. 34315433 PMC8314574

[9] K. Ahmed , R. Dasgupta , A. Vats , et al., “Urethral Diverticular Carcinoma: An Overview of Current Trends in Diagnosis and Management,” International Urology and Nephrology 42, no. 2 (2010): 331–341, 10.1007/s11255-009-9618-x. 19649767

[10] P. Khetrapal , J. K. L. Wong , W. P. Tan , et al., “Robot‐Assisted Radical Cystectomy Versus Open Radical Cystectomy: A Systematic Review and Meta‐Analysis of Perioperative, Oncological, and Quality of Life Outcomes Using Randomized Controlled Trials,” European Urology 84 (2023): 393–405, 10.1016/j.eururo.2023.08.033. 37169638

[11] G. Gakis , T. M. Morgan , S. Daneshmand , et al., “Impact of Perioperative Chemotherapy on Survival in Patients With Advanced Primary Urethral Cancer: Results of the International Collaboration on Primary Urethral Carcinoma,” Annals of Oncology 26, no. 8 (2015): 1754–1759, 10.1093/annonc/mdv230. 25969370

